# Impact of material supply chain on the productivity optimization for the construction of roads projects

**DOI:** 10.1038/s41598-024-53660-6

**Published:** 2024-02-08

**Authors:** Shrouk Awaad, Dina M. Mansour, Ibrahim Mahdi, Ibrahim Abdelrasheed

**Affiliations:** 1 Structural Engineering and Construction Management Department, Faculty of Engineering, Future University, Cairo, Egypt; 2 Structural Engineering Department, Ain Shams University, Cairo, Egypt

**Keywords:** Civil engineering, Statistics

## Abstract

Materials require special consideration when developing a project plan because they make up such a sizable chunk of the overall budget. Materials supply and delivery are crucial especially in road construction projects as they are required for the daily construction process. Lack of materials is a major source of jobsite productivity loss. This is due to the lack of structured communication and clearly defined tasks in the current materials management methods. The divergence between design and construction, the failure to coordinate and integrate multiple functional specializations, and poor communication lead to excessive fragmentation. All of these contribute to performance issues like late material ordering and delivery, low productivity, and budget overruns. This research develops a material supply chain (MSC) framework for best practices in road construction projects at all phases. This ensures that contractors receive the supplies they need at the optimum time, with the required quantities, and at the lowest possible cost. Contractors can enhance output, save money, and stay competitive. A questionnaire was designed to investigate current practices in MSC, identify the most common obstacles that faced contractors throughout the project phases, and identify the most important contributors to the integration of supply chain in construction. The developed framework was then evaluated by road construction experts; 90% stated that the proposed framework promotes project participants to share information and data. 80% assured that the framework promotes completing the project with desired quality and encourages problem solving before it even occurs.

## Introduction

Supply Chain (SC) is defined in a variety of ways^1^, the word "supply chain" (SC) refers to the interconnected network of businesses that take in raw materials, transform them into finished products, and finally ship them to customers^2,3^. A group of companies that cooperate to acquire, manufacture, and distribute a group of products that share common characteristics^4^ is another definition for SC. Supply chain management's (SCM) main objective is to make sure that the right products are accessible at the lowest feasible price in the required quantities (and at the optimum time). The goal can be broken down into sub-goals, such as adaptability, delivery consistency, and stockpile size. The reliability and timelines of deliveries are two characteristics of customer service, flexibility and inventory are also crucial^5^.

The material supply chain (MSC) is described as "a collection of enterprises that work together to bring products and services to market and into the hands of consumers." through upstream and downstream links^6,7^. It is impossible to successfully complete the project without having an effective materials management system in place. Material control is a topic that is highly significant and vital for any firm, and it is one that needs to be efficiently managed for a project to be successfully finished. Materials account for a significant portion of project costs. According to some studies, materials account for between 50 and 60 percent of project costs^8^. Materials management has many definitions as well. However, the researchers agreed that materials management is the key to project success^9^. Materials management ensures that all required materials and equipment are purchased at a reasonable price and delivered to the construction site on time without compromising quality. Materials management includes procurement, inventory, and transportation. The effectiveness of a project's budget can be improved by having a materials’ management system that is effectively organized^10^.

A well-functioning material management system can provide numerous advantages to a project^11^. Earlier research assured that labor productivity in the construction industry has increased by 6%, resulting in a 4–6% increase in savings^12^. One of these benefits is a decrease in the total amount that must be spent on materials, improving material handling, materials will be delivered on time and in the amounts necessary, improving labor productivity and project scheduling, improving supplier relations and minimizing surplus materials.

Many issues were encountered during the implementation of MSC process throughout project phases, and workable solutions were proposed. In each level of the MSCP, poor communication among the stakeholders involved was a frequent issue. The study indicated that contractors, subcontractors, and suppliers must grasp the client's goals and objectives and commit to these needs and objectives, in addition to the creation of a method for the effective management of conflicts and other challenges that may arise among the project's participants while the project is being carried out, the most important aspects that contributed to integrating the project phases and participants were creating a method for team members to effectively communicate and share relevant project information in real time^13,14^.

The construction industry is extremely fragmented, which has considerable negative effects, including productivity loss, time and cost overruns, and the claims that arise. Those have been identified as the primary causes of the industry's performance issues. Due to this degree of segmentation, the delivery process of the project is seen inefficient relative to other industries^15,16^. The construction business is plagued with numerous problems as a result of its convoluted organizational structure, which results in a broad spectrum of job opportunities, professions, and organizations form this industry^17,18^.

Because materials account for such a major percentage of a construction project's total cost as discussed earlier, extra consideration must be given to them while developing a project plan. Utilization of various kinds of materials was necessary on a daily basis for the completion of a construction project. One of the most common reasons for worksite productivity loss was a lack of materials when they were needed. The isolation of design and construction, the absence of collaboration and control across numerous functional specialties, and the inadequacy of communication all contribute to the problem, and other factors contributed to the noticed fragmentation. All of them were significant contributors to performance issues such as material receiving delays, poor productivity and time and cost overruns^19^.

Society's lifeblood is transportation and logistics. There is a substantial connection between total economic activity increase and transportation growth. Because the movement of people and commodities has the potential to generate wealth and prosperity, the quality of transportation infrastructure, particularly the condition of road networks, is frequently a governmental priority. The design, construction, and maintenance of national highways consume a significant portion of government funds. Given the size and significance of these expenditures, the timelines, efficiency, and long-term viability of these initiatives pique public interest^20^. Highway construction projects are an important element of today's investments. As a result, many researchers focused on the loss of time that happens throughout the highway construction process, notably in Egypt^21,22^.

The development of new highways, roadways, and railroads had seen a significant increase in construction during the past few years. As important as they are, it becomes more important to execute them with utmost care to optimize logistics and minimize waste. In road construction, materials play a major role as they represent more than 50% of the work required, so providing them when needed is essential in order to make the project profitable^23^. Materials deserve special consideration when developing a project plan, as they comprise a substantial proportion of the total cost of a construction endeavor. Materials play a significant role in the everyday development of construction projects. The unavailability of necessary materials is one of the leading reasons for productivity loss on a jobsite. The construction industry now uses fragmented methods of materials management, with unorganized communications and no clearly defined roles for the parties involved. The segregation of construction and design, poor communication, lack of functional discipline-wide coordination, etc. are all causes of the highly fragmented state. They are all crucial elements contributing to performance-related issues like slow material ordering and delivery, low output and budget and time overruns. A huge amount of money is consumed in these projects, which is consumed with no extra benefits as a result. Material Supply chain management is essential to resolve issues, identify and eliminate unnecessary costs, and enhance functionality and quality.

All of the previously discussed studies highlight the significance of providing the roads construction industry with a guiding tool which organizes and avoids the most common issues in the material supply chain process.

## Objectives

The main goal of this research is to develop a decision making framework for the best practices of material supply chain management (MSCM) throughout the project stages that add benefit to the road construction sector, allowing contractors to get the appropriate supplies in the right amounts, at the lowest possible cost. This will aid contractors in increasing production, reducing losses, and increasing their competitiveness. The proposed framework contributes in:

- Completing the project with the required quality.
- Implementing the project with specified time and optimum cost.
- Enhancing the collaboration among project participants.
- Sharing project information and data among parties involved.
- Stimulating resolving the problems in advance once they arise.
- Improving internal and external coordination of projects.

## Research methodology

**Figure 1:**
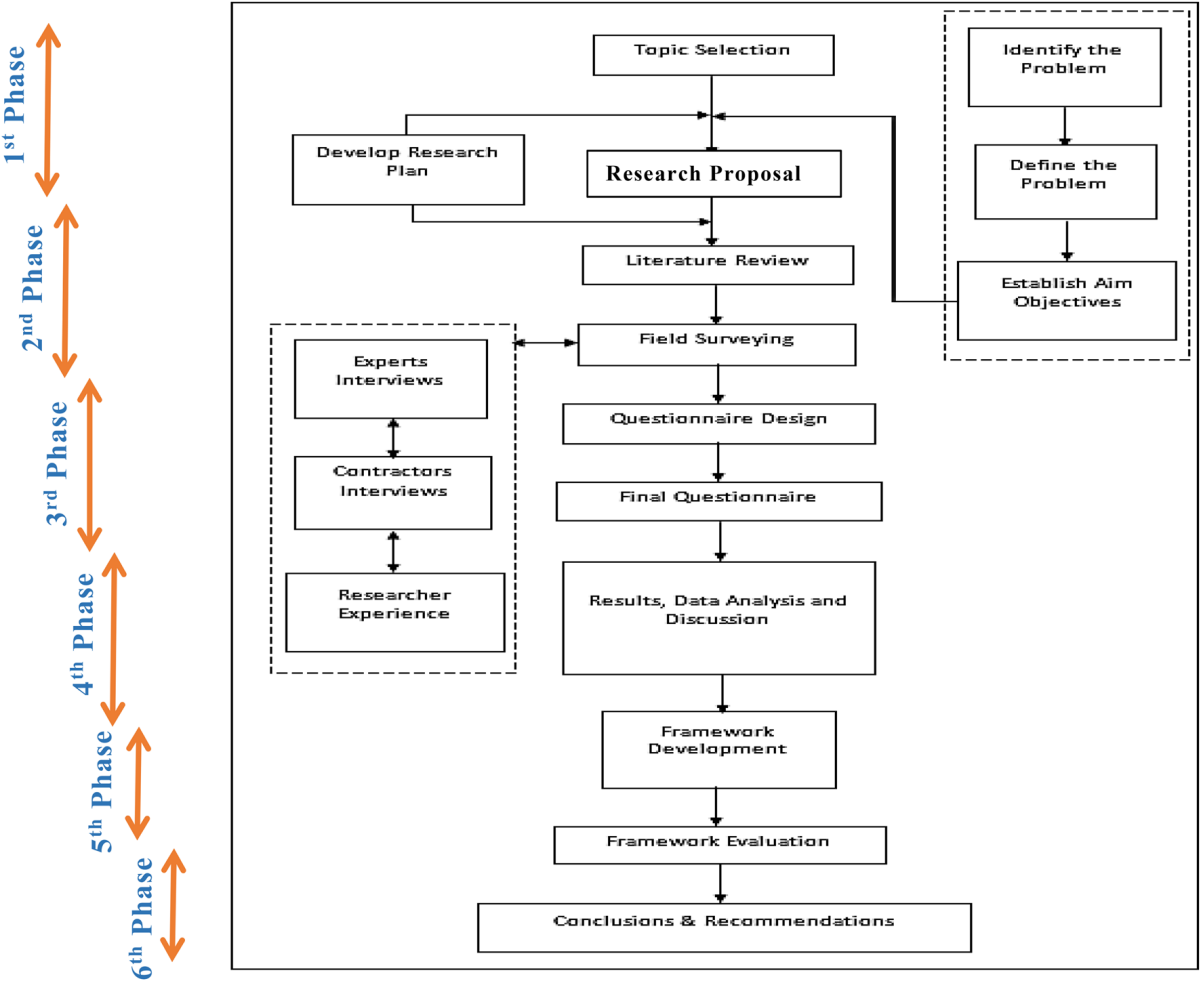
Flowchart of research methodology.

The methodology of this research can be broken down into six distinct stages as illustrated in Fig. 1. The initial part of the research involved identifying and characterizing the issues, determining the study's objectives, and designing the research strategy. Comprehensive literature review along with field study were deeply investigated in order to include the perspective of contracting businesses and all encountered issues. Then, a questionnaire was designed and distributed among roads construction companies. Statistical Package for Social Sciences (SPSS) was used in the fourth stage to analyze the data collected form the questionnaires. In the following phase, a framework of MSCP was developed based on the literature, analysis of the questionnaires, and the interviews conducted by the authors with highly experienced contractors of roads construction industry in Egypt. Eventually, the framework was evaluated by interviewing 10 contractors using the Delphi technique. The conclusions and suggestions were incorporated in the final stage as well.

## Questionnaire phase

### Questionnaire design

According to a review of published works about material supply chain management, all the information that could be helpful in attaining the study objectives was collected, examined, and formalized to be appropriate for the research questionnaire after several phases of brain storming, guidance, and amending. This was done to make the information suitable for the study survey. In-depth interviews with subject matter specialists were also undertaken by the authors of the research. All applied methods were carried out in accordance with relevant guidelines and regulations and were approved by the respondents’ companies. Informed consent was also obtained from all respondents.

To achieve the research's aim, the questionnaire was divided into four sections, as follows:

1. The first section contained company profile information.
2. The second section discussed present practices of the procedure of MSC in road construction in Egypt.
3. The third section dealt with identifying the most common problems that contractors face during the material supply chain process in each phase.
4. The primary elements that play a role in the MSCP's integration were discussed in the fourth part.

### Sample size determination

This study made use of the Yaro Yamane Statistical Formula to determine the appropriate sample size for a population that had a finite size ^24^. This strategy can only be utilized successfully if the population's total number has been ascertained. The formula can be broken down as follows:

1 $$ {\text{n}} = {\text{N}}/\left[ {1 + {\text{N}}\left( {\text{e}} \right)^{2} } \right] $$

where n = the sample size. N = the finite population. e = Tolerable error limit or the level of significance. 1 = unit or a constant.

According to the information obtained from the contractors' union, the finite population size for first class contractors ranges from 100 to 110 road companies. Using the above formula, the sample size was calculated. The sample size for this study of 105 populations with a tolerable error 0.1 was approximately 51 samples. One hundred questionnaires were distributed and 51 valid responses were collected and processed for next stages.

### Data measurement

To evaluate the factors of the questionnaire, on a five-level evaluation system, respondents were asked to rank them as follows:

The extent to which the MSCP elements were used throughout the project stages as a score of 1 to 5, with "1" indicating never and "5" indicating always. The relevance of MSCP elements as they progress through the project phases was indicated by ratings ranging from 1 to 5, with "1" being little important and "5" being very important.

The most common problems faced during the MSCP project stages, ranked from 1 to 5, with 1 indicating Never and 5 indicating Always.

The important aspects that aid in the integration of the MSCP project's stages are graded from 1 to 5, with 1 being the least important and 5 representing the most important.

These scores were then converted into significance indices using the following formula to establish the relative ordering of the factors:

2 $$ {\text{Relative}}\,{\text{Importance}}\,{\text{Index}} = \frac{\sum w}{{AN}} \times 100 = \frac{5n5 + 4n4 + 3n3 + 2n2 + 1n1}{{5N}} $$

where w is the respondent's weight (between 1 and 5) assigned to each factor. For instance, n1 would stand for the number of people who said something was not important at all, n2 for those who said it was somewhat important, n3 for those who said it was rather important, n4 for those who said it was important, and n5 for those who said it was very important. In this case, A carries the most significance (weight = 5), while the total number of respondents is represented by N. The relative importance index is a number between 0 and 1 ^25^.

### Data analysis

The quantitative statistical analysis of the questionnaire was carried out with the aid of the Statistical Package for the Social Sciences (SPSS), and the following statistical analyses were applied to the data:

1. Percentages and frequency
2. Relative Important formula
3. Mean Value

## Results and discussion of the questionnaire analysis

The results of the questionnaire responds are presented and discussed in this section. The study population's characteristics, existing practices of materials supply chain management, the most common obstacles encountered by contractors throughout the (MSCP), and the factors that may contribute to the supply chain's integration stages are all demonstrated through the next sections.

### General background and information of respondents

**Figure 2:**
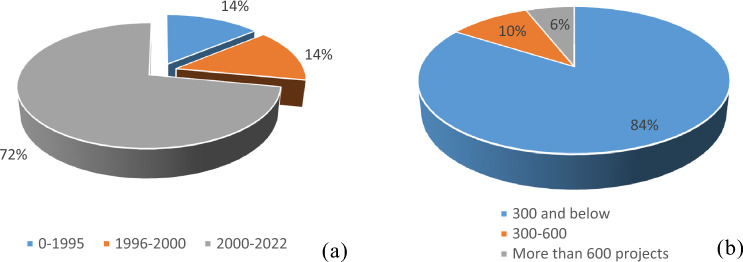
Company establishment year and number of road projects executed by the companies.

According to Fig. 2a, fourteen percent of contracting firms were established before 1995, fourteen percent between 1996 and 2000, and seventy-two percent after the year 2000 and as shown in Fig. 2b, 84% of the contractors executed less than 300 projects and 10% executed 300–600 road projects. The results showed that 6% executed more than 600 projects. The interviewed contractors are first-class with wide experience and a good reputation, as assured by the contractors’ union.

### Current practices of material supply chain process

The primary objective of this part was to research the current state of the road construction materials supply chain in Egypt and to identify the critical success criteria for the sector. Bidding, sourcing, procurement, execution, post-execution, and assessment were the six adopted stages of the materials supply chain process. In order to accomplish these two goals, the respondents were asked a series of questions and There were two primary grading systems used for these inquiries. First, the importance degree, which strived to pave the way for the development of the construction materials supply chain process, and second, the usage degree, which examined the existing material supply chain practices in road construction. Respondents were given a usage degree scale on which to indicate whether the specified procedure was always, frequently, occasionally, seldom or never at all utilized, and an importance degree scale on which to indicate whether the specified procedure was very essential, essential, quite essential, some essential, or little essential. Then, responses were analyzed for each section and importance level was obtained using the categories indicated in Table 1.

**Table 1:** Importance level categorization ^26^.

RII values	Importance level	
0.8 ≤ RII ≤ 1	High	H
0.6 ≤ RII ≤ 0.8	High–Medium	H–M
0.4 ≤ RII ≤ 0.6	Medium	M
0.2 ≤ RII ≤ 0.4	Medium–Low	M–L
0 ≤ RII ≤ 0.2	Low	L

#### Bidding phase

This sector comprises nine components that comprised the MSCP's bidding process. The respondents were asked how frequently they used these items and how important they thought they were. The findings are demonstrated in Table 2, which shows that 89% of the suggested components are highly important. This is because the bidding phase is extremely important for contractors in order to be awarded the contract with a reasonable margin of profit.

**Table 2:** Bidding process phase.

Item no	Material supply chain process bidding process phase	Usage degree			Importance degree		
		Mean	RII	Importance Level	Mean	RII	Importance Level
1.1	Identify the materials that are required for each item	4.50	0.9	H	4.38	0.876	H
1.2	Quantity survey for the needed materials	4.62	0.924	H	4.56	0.912	H
1.3	Creating a price database for materials from previously projects to be utilized in producing estimates for future projects	3.53	0.702	H-M	3.57	0.711	H-M
1.4	On preparing the project estimate, consider the prices of suppliers and manufacturers	3.94	0.788	H-M	4.04	0.808	H
1.5	Checking the estimate's prices before submitting a bid	4.62	0.924	H	4.5	0.9	H
1.6	You should hold a meeting with the workers and the project manager as soon as you find out that you have won the bid so that you can adjust the initial estimate of the project's scope	4.46	0.892	H	4.26	0.852	H
1.7	Producing a preliminary material requisition schedule that includes type of materials, quantity required and when the material should be supplied in jobsite	4.48	0.896	H	4.46	0.892	H
1.8	Special materials that need prefabrication or special suppliers	4.02	0.804	H	4.1	0.82	H
1.9	Prepare the estimate using the use of a computer programmer, such as Microsoft Excel	4.97	0.970	H	4.92	0.983	H

#### Sourcing phase (supplier selection)

**Figure 3:**
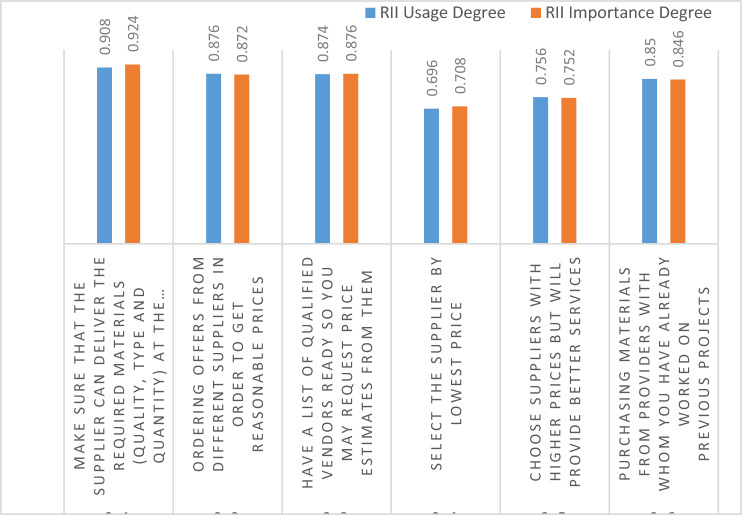
Sourcing phase.

The sourcing phase of the MSCP was comprised of six components. The participants were asked how frequently they used these items and how important they thought they were. The findings are summarized in Fig. 3. 67% of the components were highly important, as the contractors are selecting suppliers who guarantee the availability of the required quantity of materials, so giving the lowest score to suppliers with lower prices.

#### Procurement phase

**Figure 4:**
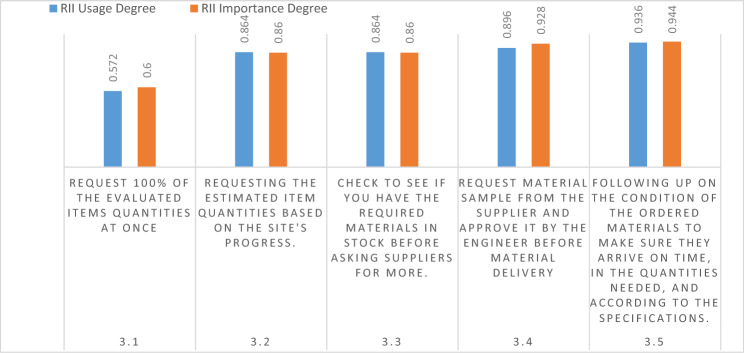
Material procurement process.

This sector covers five items that comprised the MSCP's procurement phase. The participants were asked how often they used these items and how important they thought they were. The outcomes are shown in Fig. 4; it shows that most contractors do not rely on the preliminary quantity surveying as they do not request 100% of the item quantity at once, which was considered the least important item. This is a major flaw, as ordering supplies in smaller quantities naturally comes at a higher cost per unit than bulk materials, which further escalates the total cost.

#### Execution phase

**Figure 5:**
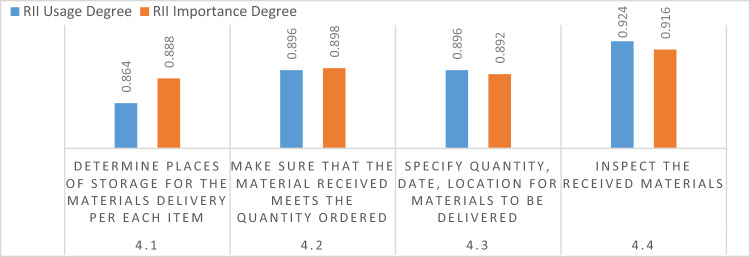
Execution process phase.

Four items were discussed and analyzed for the execution phase as presented in Fig. 5. In this phase, all the items mentioned were given a high importance ranking. Storage area, received quantity, delivery instructions, and inspection of materials are all crucial and key factors for successful projects.

#### Post execution phase

One essential component of the supply chain in the road constructing business is minimizing the amount of material excess to the greatest extent possible. This component is directly connected to the quantity characteristic. In this section, four potential ways to dispose any remaining materials when a project is completed is presented. Participants were given a list of possible situations and asked to indicate how often they had experienced each one. The results are shown in Table 3. The results show that 54% of the respondents keep the excess materials for future projects, which highlights the importance of conducting an accurate and precise preliminary quantity survey to avoid facing these situations. This causes material waste, which is pricey for construction projects due to paying money for unneeded materials and the accompanying storage and disposal costs. Material waste can also arise from deterioration in storage, either due to improper storage conditions or expiring before being used.

**Table 3:** Post execution phase (surplus materials).

Item no	Post construction phase	% of occurrence	Rank
5.1	Keeping the excess materials for future projects in storage	54.2	1
5.2	Return surplus materials to suppliers without loss	23.7	2
5.3	Selling the surplus materials to other construction companies	19.8	3
5.4	Return surplus materials to suppliers with penalty	2.3	4
	Total	100%	

#### Assessment phase

In this phase, the respondents were questioned how they use and value the MSCP throughout the project phases. The outcomes are summarized in Table 4. The contractors assured that performing a comprehensive assessment of the material supply chain technique throughout the phases to reduce errors and enhance the procedure for future projects is extremely important, but unfortunately, they disclosed not following this procedure in most cases.

**Table 4:** Assessment phase outcomes.

Item no	Assessment phase	Usage degree			Importance degree		
		Mean	Relative index	Importance level	Mean	Relative index	Importance level
6.1	Performing a comprehensive assessment of the material supply chain technique throughout the phases to reduce errors and enhance the procedure for future projects	3.42	0.68	M	4.12	0.824	H

### Obstacles that faced contractors during material supply chain process

Understanding the issues at hand is essential for effective problem solving. Many issues may arise for contractors during the procedure of material supply chain, obstructing the achievement of supply chain management's primary goals. This sector seeks to identify the most common issues contractors faced during the material supply chain process, which is divided into five phases: bidding, sourcing, procurement, execution, and post-execution. The sector also investigated the core causes of those issues and proposed potential solutions in the developed framework in order to ensure that the MSCP application runs smoothly and without interruptions. The results are shown in Table 5.

**Table 5:** Problems faced contractors during the different phases.

No	Problems	Degree of occurrence		
		Mean	Relative index	Rank
1	Bidding phase			
1.1	Poor communication between the involved parties	3.8	0.76	1
1.2	Variations between designs and specifications	3.3	0.66	2
1.3	Incomplete drawings and missing details	3.1	0.62	3
2	Sourcing phase (supplier selection)			
2.1	Incomplete proposals from suppliers	3.24	0.648	1
2.2	Having a large number of suppliers and having no data about them	3.04	0.608	2
3	Procurement phase			
3.1	Not enough money on hand	3.8	0.76	1
3.2	Unavailability of required materials due to increased demand	3.18	0.636	2
4	Execution phase			
4.1	Late deliveries of required materials	3.22	0.644	1
4.2	The materials that were given do not meet the necessary requirements	3.07	0.614	2
4.3	Poor communication between the parties involved	3.06	0.612	3
4.4	Storage area of materials is far away from the jobsite	2.74	0.548	4
4.5	Theft	2.18	0.436	5
5	Post-construction phase			
5.1	No possibility for the excess materials to be returned back to the supplier	3.32	0.664	1
5.2	No warehouses for the extra materials	3.02	0.604	2
5.3	Salvage losses for the surplus materials	2.7	0.54	3

### Key factors contributing to the integration of supply chain

The integration of the owner, general contractor, subcontractors, and suppliers, as well as the integration of the project various phases, will help the MSCP to run progress smoothly through the phases of projects. The key elements that can help the MSCP's for all project phases to come together are presented in this section. Nine factors were addressed and each one was rated by the respondents as extremely important, important, quite significant, some important, or not important at all. The outcomes are shown in Table 6. The respondents assured the necessity of developing a framework for accurate information sharing and communication among project participants (Relative index = 0.91).

**Table 6:** Factors that could help the process of supply chain integrate successfully.

Item no	Factors	Mean	Relative Index	Rank
6.1	Contractors, suppliers, and subcontractors should commit to understanding and supporting the client's demands and objectives	4.83	0.97	1
6.2	The use of (design-build) contract between the contractor and the customer	3.88	0.7	9
6.3	Designers should complete their participation until construction phase	4.32	0.86	5
6.4	Facilitating a supplier and subcontractor workshop to address concerns with quality, progress, and safety	3.88	0.78	8
6.5	Establishing a framework for timely and accurate information sharing and communication among project participants	4.56	0.91	2
6.6	Establishing a procedure for handling conflicts and issues that may occur amongst project participants while the project is being executed	4.34	0.87	4
6.7	Aligning your company's system and procedures with those of your client, suppliers, and subcontractors	4.38	0.88	3
6.8	rather than using a competitive tendering process, negotiating contracts with suppliers and subcontractors	4.26	0.86	6
6.9	Accessing and sharing project-related information via a web-based system	3.99	0.79	7

## Proposed framework for material supply chain process in road construction

The primary goal of the suggested framework was to make it possible for construction companies to provide the proper materials, in the necessary quantities, at the optimum time, and for the lowest possible cost by managing the supply chain throughout the phases of a project as a comprehensive procedure strategy instead of a collection of discrete activities. All of this will eventually enhance productivity consequently.

The framework is based on the findings of the questionnaire and a review of the literature. SCOR model is one of the most popular supply chain managements (SCM) reference models^27^. The Supply Chain Council, an esteemed international non-profit organization, developed this model to assist with SCM by providing criteria for judging SCM strategies^28^. Plan, source, make, deliver, and return are the five cornerstones of the SCOR framework. The elements represent the primary inter-organizational processes that occur throughout a product's entire lifespan^28,29^. Typically, the SCOR model was used to optimize SC processes by discovering, analyzing, and reorganizing them.

The framework introduces an overview of the various and most significant and influential elements found in the SCM literature and through the interviews and the questionnaires responses analysis and how they relate to each other. The framework's goal is to handle all phases of roads construction projects, address the most frequent challenges contractors confront, introduce alternative solutions, and ultimately integrate elements of the material supply chain process. The proposed framework is intended to provide an overview of the numerous SCM components included in the research as well as their interrelationships.

**Figure 6:**
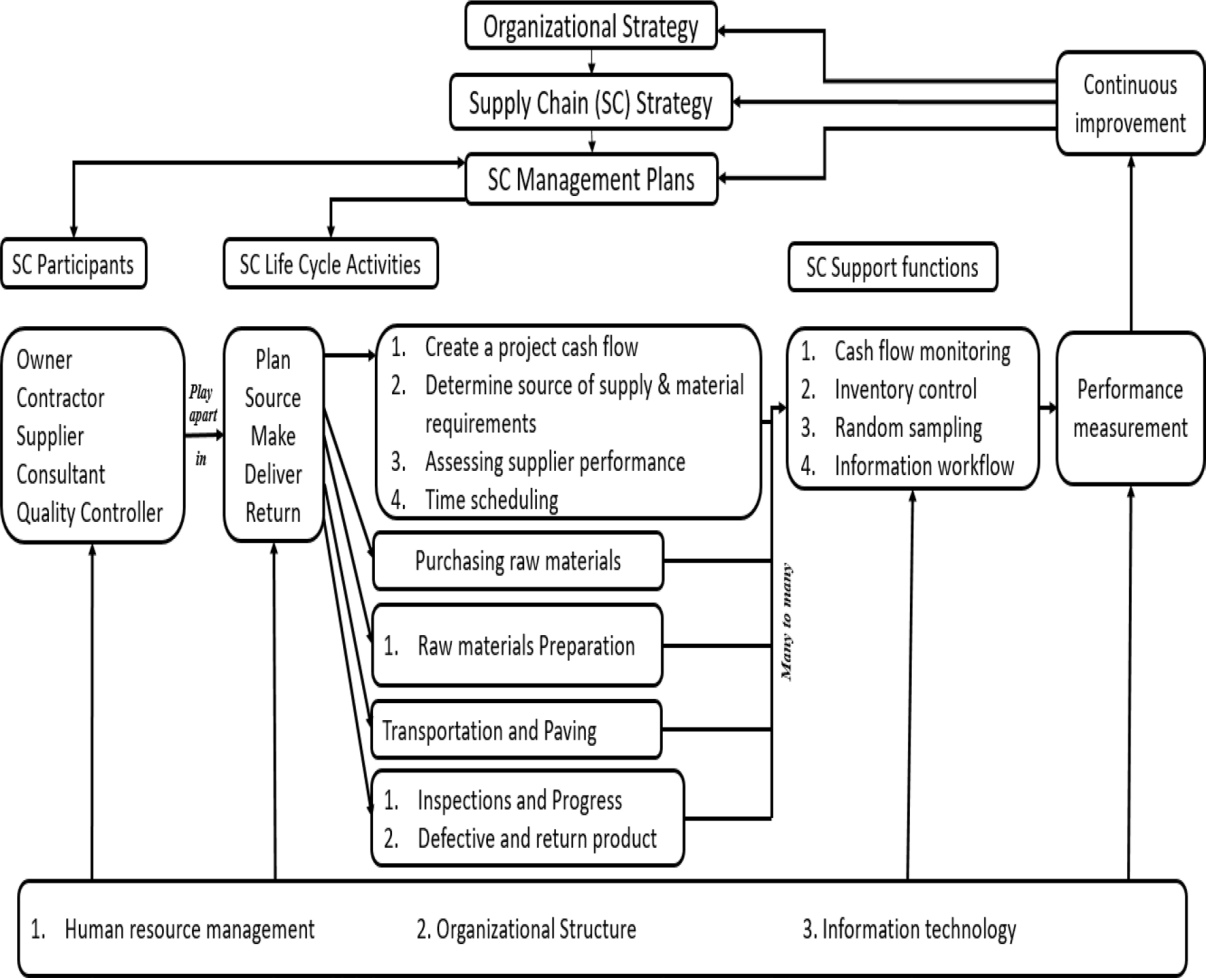
The proposed material supply chain framework.

The SC was divided into three categories: SC life-cycle activities, SC participants, and SC support activities using themes as shown in Fig. 6. The proposed SCM framework has identified the subcomponents within each of its primary components using the SCOR model. Participants are all the major characters who could have an impact on a company's SC. The primary procedures used to convert raw materials into finished goods are called life-cycle activities. Supporting tasks are those that connect to various SC activities and are used to guide and control the SC.

The developed SCM framework in Fig. 6 begins with organizational plan and flows into SC plan as it is critical to align SC strategy with organizational strategy. Management plans are used to implement strategies. SCM also has three major constituents: SC life-cycle activities, SC participants and SC support functions. SC participants are connected to SCM plans and are involved in life-cycle activities. The SC support functions and SC life-cycle activities have a many-to-many relationship. Performance measurement is an important component of the proposed framework because it serves as a feedback loop for nonstop enhancement, influencing management plans and supply chain strategy. Supply chain enablers that act across functions, activities, and participants have an impact on each of the SCM components. The relative positions of the objects and the direction of the flow between them, as denoted by arrows, show the relationships between the components.

### Organizational strategy, supply chain strategy, and supply chain management plans

As can be seen in Fig. 6, the organizational strategy sits atop the suggested SCM structure. Some authors use the phrases "corporate strategy" and "organizational strategy" interchangeably. Andrews argues that whereas a "business plan" may be applicable to a single product line or department, "corporate strategy" encompasses the entire corporation^30^. Engineers frequently mix up the terms "SC strategy" and "SCM." While SCM more specifically refers to the interplay between various supply chain activities, supply chain strategy specifies how a firm should be established and run in order to stand out^27^.

### Participants

SCM participants are entities or individuals that take part in or are participating in a certain SC. SC is termed multi-disciplinary because it has numerous role players from various disciplines. According to an organization's SC scope and its outsourced functions, some participants also come from within the organization, while others do so from other organizations. The role-players in a generic SC are divided into the participant component for the purposes of the suggested framework in Fig. 6: Owner, contractor, Supplier, consultant and quality controller.

### Supply chain life-cycle activities

SC activities include all tasks that have an effect on products at any point during their life cycle, beginning with the supplier (who is responsible for the raw material) and ending with the consumer (who is in possession of the finished product). For the suggested structure, activities are referred to as life-cycle activities, and the SCOR model is used to classify them according to the following headings: plan, source, make, deliver, and return.

The "plan" process involves activities including creating a project budget, determining supply resources and material requirements, evaluating supply resources, Time scheduling and arranging transportation. The "source" processes cover all actions involved in getting, receiving, buying, and paying for raw materials. The "make" process involves procedures including ordering and receiving resources, producing, fabrication and testing. The "deliver" procedure covers all interactions between the producer and the consumer, including order management procedures and transportation. The 'return' procedure comprises all defected and returned products handling, inspections, and credit note invoicing^27^.

### Supply chain support functions

The fundamental life-cycle processes of a SC are controlled and supported by a variety of management functions. The suggested SCM framework refers to these tasks as support functions. Numerous support functions in the field of SCM can be listed indefinitely. For the sake of this study, the framework only includes a subset of the typical operations described in the SCM literature. This is by no means an exhaustive survey of SCM's support roles; rather, it serves to illustrate some of the ways in which issues along the supply chain could be addressed. The subcomponents included in the suggested framework are Inventory Control, taking random samples to ensure specifications and accuracy, cash flow monitoring and information workflow.

### Performance measurement and continuous improvement

Recently, measuring organizational performance has attracted a lot of attention. Metrics and indicators of performance are critical to an organization's success because of the influence they have on all aspects of the planning and management processes^31^. The feedback loop that connects SC management plans, SC strategy, and SC support functions is one of the components that the proposed framework includes for measuring performance. In the context of dynamic SC, the issue of continuous improvement is quickly becoming one of the most important ones. Suppliers, owners, and contractors all collaborate on efforts to continuously improve their businesses in order to gain a competitive advantage^32^.

### Supply chain management enablers

Participants, life-cycle activities, support functions, and performance measurement are all impacted by aspects that enable their performance in the context of the suggested framework. These components are known as 'supply chain enablers.' as with support functions, there are numerous enablers in the context of SCM. Human resource management, organizational design and information technology are seen as essential enablers of effective SCM in this article.

Human resources are the most important supply chain enabler as any company's success depends on how well-trained its staff is. Organizational design is the process of evaluating and deciding on the formal structure and system of communication, labor division, coordination, control, leadership, and responsibility necessary to accomplish organizational goals and objectives.

In the twenty-first century, there has been a significant increase in the development of information technology (IT) platforms and applications that support an end-to-end supply chain. Supply chain planning and execution are the two main supply chain apps used in purchasing-related supply chain collaboration.

## Evaluation of the proposed framework for material supply chain process in road construction in Egypt

**Figure 7:**
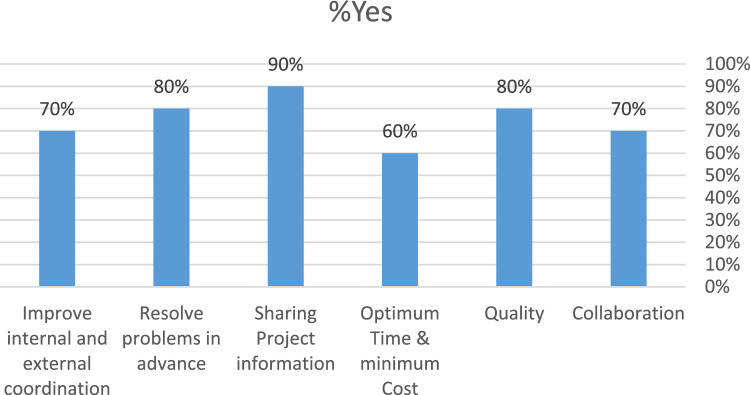
Percentage of yes response to the feedback questionnaire of the proposed framework.

The Delphi technique was used in conjunction with a face-to-face survey questionnaire to evaluate the developed framework. This feedback form utilized the Delphi technique, which has been shown to be a successful method for gathering information from experts in a particular field of knowledge^33,34^. Delphi technique is based on performing multiple iterations (rounds) to collect responses^35–37^. Ten contractors with relevant experience were visited, and the authors explained the framework. Then, a feedback questionnaire was distributed, and the experts were asked to answer with Yes or No for each of the listed questions. The results of the questionnaire are presented in Fig. 7. The questionnaire is administered in up to two rounds, with the same results in each.

## Conclusions and recommendations for future works

The following are the main conclusions that can be drawn from this research:

It was discovered that the most frequently encountered problems by contractors were:

*In Biding phase*

- Poor communication between the involved parties.
- Differences between specifications and designs.
- Incomplete drawings and missing details.

*In Supplier Selection phase*

- Missing proposals (Suppliers may not provide the whole set of documentation with the proposal).

*In Material Procurement Phase*

- Not enough money on hand.
- Unavailability of required materials due to increased demand.

*In Execution Phase*

- Late deliveries of required materials (Materials do not arrive as scheduled).
- The delivered materials do not meet the specifications.

*In Post Construction Phase*

- No possibility to return surplus materials to the supplier.
- No storage places for surplus materials.

- The most essential component that could aid in integrating the MSCP project stages is the contractors, subcontractors, and suppliers' understanding of the client's goals and objectives and establishing a framework for timely and accurate information sharing and communication among project participants.
- It was found that it is essential to create a system for project participants to communicate and share project information promptly and accurately.
- The proposed framework promotes project participants to share information and data as 90% of the responded contractors stated that.
- 80% of the responded experts stated that the suggested framework promotes completing the project with desired quality.
- 80% of the survey respondents agreed that the introduced framework encourages problem solving before it even occurs.

In order to cover more areas of the topic in this research, Additional topics might be investigated:

1. Extending the current research to include case studies.
2. Upgrading the proposed framework to include key performance indicators.
3. Developing a website as an integrated project planning tool.
4. Researching to study management of inventories.
5. Studying Lean and Six Sigma process improvement projects that had been used to assist in the on-going process of Supply Chain improvement.

## Data Availability

All data is introduced and discussed through the article.
